# Variability in protein cargo detection in technical and biological replicates of exosome-enriched extracellular vesicles

**DOI:** 10.1371/journal.pone.0228871

**Published:** 2020-03-02

**Authors:** Suma Tiruvayipati, Don Wolfgeher, Ming Yue, FangFang Duan, Jorge Andrade, Hui Jiang, Lucia Schuger

**Affiliations:** 1 Biological Sciences Division, Department of Pathology, The University of Chicago, Chicago, Illinois, United States of America; 2 Proteomics Core Laboratory, Cummings Life Science Center, The University of Chicago, Chicago, Illinois, United States of America; 3 The Center for Research Informatics, The University of Chicago, Chicago, Illinois, United States of America; 4 Department of Biostatistics, University of Michigan, Ann Arbor, Michigan, United States of America; NIH, UNITED STATES

## Abstract

Exosomes are extracellular vesicles (EVs) of ~20–200 nm diameter that shuttle DNAs, RNAs, proteins and other biomolecules between cells. The large number of biomolecules present in exosomes demands the frequent use of high-throughput analysis. This, in turn, requires technical replicates (TRs), and biological replicates (BRs) to produce accurate results. As the number and abundance of identified biomolecules varies between replicates (Rs), establishing the replicate variability predicted for the event under study is essential in determining the number of Rs required. Although there have been few reports of replicate variability in high throughput biological data, none of them focused on exosomes. Herein, we determined the replicate variability in protein profiles found in exosomes released from 3 lung adenocarcinoma cell lines, H1993, A549 and H1975. Since exosome isolates are invariably contaminated by a small percentage of ~200–300 nm microvesicles, we refer to our samples as exosome-enriched EVs (EE-EVs). We generated BRs of EE-EVs from each cell line, and divided each group into 3 TRs. All Rs were analyzed by liquid chromatography/mass spectrometry (LC/MS/MS) and customized bioinformatics and biostatistical workflows (raw data available via ProteomeXchange: PXD012798). We found that the variability among TRs as well as BRs, was largely qualitative (protein present or absent) and higher among BRs. By contrast, the quantitative (protein abundance) variability was low, save for the H1975 cell line where the quantitative variability was significant. Importantly, our replicate strategy identified 90% of the most abundant proteins, thereby establishing the utility of our approach.

## Introduction

Extracellular vesicles (EVs) include microvesicles and exosomes[1–3]. Exosomes are bilayered membrane-bound nanovesicles [4] of endocytic origin, ranging from ~20 to 200 nm[3, 5, 6], emerging during the formation of multivesicular bodies and secreted into the extracellular space as a result of the fusion of multivesicular bodies with the plasma membrane [7, 8]. Exosomes have specific surface proteins such as flotillin-1 and tetraspanin family proteins CD9, CD81, and CD63 [7, 9]. In addition, membrane proteins such as annexins and components of the ESCRT complex, and integrins were also characterized in these EVs [10]. Exosomes are constantly released by all types of cells, normal or diseased, and are present in all body fluids [7]. They contain DNA, RNAs, proteins, and lipids [11, 12] and provide local and distal biological signals to tissues via endocytic transfer of their contents [11].

Exosomes participate in multiple normal biological processes [11] and play a significant role in a myriad of pathological conditions such as cancer progression, autoimmune and infectious diseases, obesity, and neurodegenerative diseases [11]. Since exosomes reflect the phenotype of their donor cells [2, 13–15], they became an important part of the newest repertoire of what is referred as the “tumor circulome” in liquid biopsies with a promising potential in cancer management [16]. Moreover, exosomes are being studied as agents for gene therapy, vaccines, and drug delivery [2, 4].

The Exocarta (http://www.exocarta.org/) database, incepted in 2009, compiles the RNAs and proteins from a wide range of exosomal data [17]. Following which, EVpedia (http://evpedia.info) an integrated proteome, transcriptome, and lipidome database has led to considerable improvement in EVs research [18].

It remains worthy to point to the existence of technical caveats in exosome research, as currently all the conventional methods of exosome isolation pervasively retain a small percentage of ~200–300 nm microvesicles [6, 19, 20]. Therefore, research is now being conducted to obtain pure types of EVs [3]. Due to this well-established factor, we refer to the exosome isolates obtained by current techniques as exosome enriched EVs (EE-EVs).

Due to the large biomolecular cargo carried by these EVs, exosomal research often relies on the generation of high-throughput data. A proper interpretation of data generated by high-throughput analysis requires the use of replicate samples (Rs). These include technical replicates (TRs) and biological replicates (BRs). TRs help to understand the reproducibility of an assay, whereas BRs inform about the reproducibility of the phenomenon [21]. Therefore, both have to be included in the design of any experiment in order to reach accurate conclusions.

As variability in the number and abundance of identified biomolecules (here respectively referred to as qualitative and quantitative variability) is always encountered among Rs, it is essential to know the variability in sampling expected for the specific phenomenon under study in order to determine the number of Rs adequate to generate accurate results.

Although there have been few reports of Rs variability in high throughput biological data [22–24], none of them focused on exosomes. Therefore, here we used custom bioinformatics and biostatistics workflows of LC/MS/MS data to determine the qualitative and quantitative variability in proteins in TRs and BRs of EE-EVs from lung adenocarcinoma cell lines.

## Materials and methods

### Cell culture

**Fig 1:**
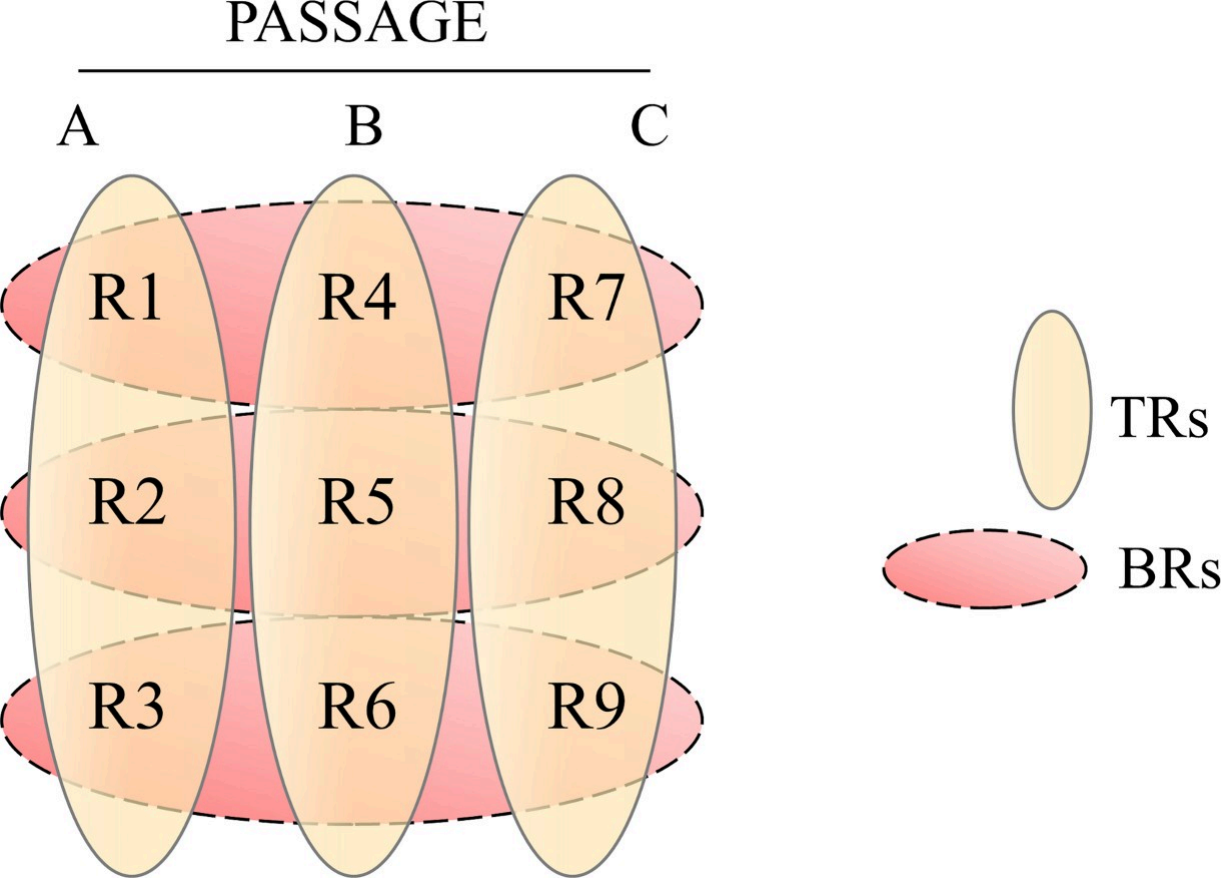
Schematic representation of exosomal samples collection. Exosomes were collected from three different cell lines at three different passages (A, B, C) to provide three biological replicates (BRs). The exosome lysate from each of them was further divided into three technical replicates (TRs). The nine samples were grouped so that the TRs were TR1: R1, R2, R3, TR2: R4, R5, R6, TR3: R7, R8, and R9. The BRs were BR1: R1, R4, R7, B2: R2, R5, R8, BR3: R3, R6, and R9.

The cells used in the current study were human lung adenocarcinoma cell lines: H1993 (ATCC CRL-5909), H1975 (ATCC CRL-5908) and A549 (ATCC CCL-185). H1975 and H1993 cells were cultured in RPMI media (Gibco, 11875119) while, A549 cells were cultured in Ham’s F-12K media (Corning, 10-025-CV) with 100 Units/ml penicillin– 100 μg/ml streptomycin (Gibco, 15140122) and 0.25 μg/ml amphotericin (Gibco, 15290026). Media was supplemented with 10% fetal bovine serum (FBS) (Atlanta biologicals, S11150). Cells were grown to 85–95% confluency in 8 150 mm cell culture dishes (Nunc^TM^, 157150), trypsinized (using 0.25% trypsin EDTA (Gibco, 25200114)), counted with a hemocytometer and seeded in 3 corning 224 mm cell culture dishes with 10% exosome depleted FBS (SBI, EXO-FBS-250A-1). Cells were incubated at 37°C and 5% CO_2_ and after 24 hours, the medium was collected for exosome purification. Triplicate exosome enriched extracellular vesicles (EE-EVs) samples from each of the 3 lung adenocarcinoma cell lines H1993, A549 and H1975 generated at different passages were used to generate 3 technical replicates (TRs) and 3 biological replicates (BRs) (Fig 1). In total, 9 replicate samples (Rs) per cell line that is, 27 Rs in total were analyzed for this study. The 9 samples were grouped so that the TRs were TR1: R1, R2, R3, TR2: R4, R5, R6, TR3: R7, R8, and R9. The BRs were BR1: R1, R4, R7, BR2: R2, R5, R8, BR3: R3, R6, and R9.

### Exosome enriched extracellular vesicles (EE-EVs) purification

Cell debris from the media was removed by centrifugation at 300 x g for 5 minutes followed by 3,000 x g for 45 minutes. Care was taken not to touch the pellets. As an additional quality control measure, the portion of the media touching the pellet was discarded to avoid contamination. The supernatant was transferred to 50 ml centrifuge tubes and mixed with exosome precipitation solution exoquick-TC (SBI, EXOTC50A-1) [25–27] at a 5:1 ratio and incubated for 16 hours at 4°C to precipitate exosomes followed by centrifugation at 1,500 x g for 60 minutes to collect the exosome pellets. The supernatant was discarded. The exosomes were then lysed using RIPA buffer (Thermo Fisher Scientific, PI89900) with protease and phosphatase inhibitors (Pierce, A32961) and the protein concentration was measured by Pierce BCA protein assay kit (Thermo Fisher Scientific, PI23227). Western blots were performed with cells and EE-EVs lysed in RIPA lysis buffer (Sigma-Aldrich) containing proteinase inhibitors (Roche). Lysates were cleared by centrifugation for 10 minutes at 12,000 rpm and supernatant fluids were collected. Immunoblots were performed as previously described [28]. The following antibodies (Abs) were used for immunoblotting: anti-CD 81, anti-CD 63, anti-CD 9 (all from System Biosciences), anti-Flotillin 1, anti-TSG 101, and anti-Calnexin (all from Abcam). All Abs were used at the dilutions recommended by the manufacturers. Further, the diameter of the isolated vesicles was determined using the qNano-Tunable Resistive Pulse Sensing (TRPS) at Izon Science, USA. EE-EVs were analyzed by nanopore NP150, which has a pore size of 150 mm at 5 mbar pressure. Data acquisition and analysis were performed using the Izon Control Suite software version 3.3.2.2001. The reason for electing this method is because TRPS has the advantage of detecting EVs at a higher precision than other methodologies [29–31].

### Proteomic analysis

A 100 μg of total protein of exosomes was heated with 4x SDS loading buffer (Thermo Fisher Scientific, NP0007) for 10 minutes at 70°C and loaded on 4–12% Bis-tris protein gels (Thermo Fisher Scientific, NP0335). Gels were then prefixed in 1:2:1 methanol, acetic acid, and water overnight followed by staining with Brilliant blue G solution (Sigma, B8522) for 2 hours and further destained in 10% acetic acid for 4 hours. The protein bands were then excised, placed in 1.5 ml individual centrifuge tubes with 100 μl 5% acetic acid and sent for liquid chromatography-mass spectrometry (LC/MS/MS) performed at the Michigan State University (MSU) Proteomics Core Facility. The experimental protocol was as follows: Gel bands were digested in-gel according to a previously reported study [32]. Briefly, the gel bands were dehydrated using 100% acetonitrile (ACN) and incubated with 10 mM dithiothreitol in 100 mM ammonium bicarbonate, pH~8, at 56°C for 45 minutes. The gel bands were dehydrated again in 100% ACN to force out all aqueous buffers and allow the addition of 50 mM iodoacetamide in 100 mM ammonium bicarbonate to equilibrate all the protein and incubated in the dark for 20 minutes. The gel bands were then washed with ammonium bicarbonate and dehydrated again in 100% ACN followed by an overnight incubation at 37°C with sequencing grade modified trypsin (Promega, V5111) prepared in 50 mM ammonium bicarbonate and added at ~1:50 ratio. Peptides were then extracted from the gel by water bath sonication in a solution of 60% ACN and 1% trifluoroacetic acid (TFA) and vacuum dried to ~2 μL. Dried peptides were then re-suspended in 2% ACN/0.1% TFA to 25 μL. From this, 5 μL was automatically injected by a Thermo EASYnLC 1000 liquid chromatography system onto a Thermo Acclaim PepMap 100 C18 trapping column (0.1 mm x 20 mm, 5 μm, 100A) and washed with buffer A (99.9% water/0.1% formic acid) for ~5 minutes. Bound peptides were then eluted onto a Thermo Acclaim PepMap RSLC C18 resolving column (0.075 mm x 500 mm, 3 μm, 100A) for over 125 minutes with a gradient of 5% buffer B to 28% buffer B (99.9% ACN/0.1% formic acid) for 114 minutes, ramping to 90% buffer B at 115 minutes and held at 90% buffer B for the duration of the run at a constant flow rate of 300 nl/min.

Eluted peptides were sprayed into a Thermo Fisher Q-Exactive mass spectrometer using a FlexSpray nano-spray ion source. Survey scans were taken by the ion trap, a second mass analyzer of the mass spectrometer i.e. the Orbitrap (70,000 resolutions, determined at m/z 200). In each survey scan, the top ten most intense peptide ions were automatically selected and subjected to higher energy collision induced dissociation with fragment spectra acquired at 17,500 resolutions. Conversion of MS/MS spectra to peak lists was done using Mascot Distiller version 2.6.1 (www.matrixscience.com). Peptide-to-spectrum matching was done using the Mascot search algorithm version 2.6, against a database containing all human protein sequences available from UniProt (www.uniprot.org, downloaded on 11-13-2017) and appended with common laboratory contaminants (www.thegpm.org). The search output was then analyzed using Scaffold Q+S version 4.8.4 (www.proteomesoftware.com) to probabilistically validate protein identification and quantification. Assignments validated using the default confidence filter of 1% False Discovery Rate (FDR) at the protein level in order to allow maximum discovery at reasonable stringency were considered true.

Mascot parameters for all databases were as follows: allow up to 2 missed tryptic sites, fixed modification of carbamidomethyl cysteine, variable modification of oxidation of methionine, peptide tolerance of +/- 10 ppm, MS/MS tolerance of 0.3 Da, peptide charge state limited to +2/+3.

The mass spectrometry proteomics data have been deposited in the ProteomeXchange Consortium via the PRIDE [33] partner repository with the dataset identifier PXD012798 and 10.6019/PXD012798.

### Bioinformatics analysis

#### Protein identification

Label-free quantitative (LFQ) intensity values were generated with the tool MaxQuant (version 1.6.0.0) [34] (www.biochem.mpg.de/5111795/maxquant) using “.raw” files provided by the MSU proteomics facility and searching against a Uniprot human database (downloaded on 2/1/2018). The parameters in MaxQuant were set as follows: oxidation of methionine and protein N-terminal acetylation were allowed as variable modifications, and cysteine carbamidomethyl was set as a fixed modification. The option for proteases was chosen as trypsin/P (proline) [35] which marks the cleavage at the carboxyl side of the lysine and arginine amino acids with 2 missed cleavages allowed. The parameter for label-free modification to check for protein presence was selected as LFQ values. The FDR with a *p*-value less than 0.01 was determined as significant. For protein quantification firstly, label minimum ratio count was set to 2, secondly, both unique and razor peptides were selected, and thirdly, the modifications were once again set to oxidation of methionine and protein N-terminal acetylation, along with their unmodified peptides. The obtained proteingroups.xlsx output file was sorted by descending LFQ values and used as an LFQ value reference list for further bioinformatics analysis in addition to which the LFQ values were processed with custom shell scripts for further biostatistical analysis of qualitative data.

#### Protein quantification

Absolute protein expression (APEX) values were generated using “protXML” files, a required file format by the APEX tool [36, 37]. For this, “.sf3” files provided by the MSU proteomics facility were processed to generate “protXML” files using the Scaffold 4.8.4 [38] (www.proteomesoftware.com) software, set with a cut off corresponding to a peptide and protein FDR corrected *p*-value of less than 0.01. The peptide FDR was calculated by the tool as a percentage of the sum of the exclusive spectral counts of decoy proteins divided by the sum of exclusive spectral counts of target proteins. The protein FDR was calculated as a percentage of the number of decoy proteins divided by the number of target proteins. The “protXML” files and the Uniprot human database (downloaded on 2/1/2018) was used to calculate the APEX values by following the apex protocol [36, 37] using the tool APEX_1_1_0 (https://sourceforge.net/projects/apexqpt/). The top 50 proteins from the LFQ intensity list from MaxQuant were considered to build a reference list for use with the APEX tool to generate an “.apex” file. The protein’s abundance was usually presented as *relative* to all protein within the sample, here the multiplicative normalization factor C, which multiplies the protein’s abundance by C, places the abundance values into *absolute* terms where C corresponds to “1.0E8”. The “.apex” file was further processed with custom shell scripts to proceed with biostatistical analysis. The absolute counts obtained in an “.apex” file are directly proportional to the protein levels, and used for biostatistical analysis of quantitative data.

The LC/MS/MS spectra database matching identifies peptides, and not proteins [39]. Hence, the protein list reported by LFQ values is only tentative, as several peptides can be assigned to more than 1 protein [39]. For an absolute quantitative count, the APEX proteomics tool [37] was used, which calculates abundances of protein expression based upon machine learning correction factors, LC/MS/MS spectral counts, and correct identification of protein probability. Hence, the protein list reported by APEX values is more reliable in view of identification of a complete protein sequence.

### Biostatistical analysis

Statistical analysis was performed by using the LFQ (protein identification) and APEX (protein abundance) values of LC/MS/MS data. The LFQ values were used to perform statistical analysis to show the qualitative variability while, the APEX values were used to perform statistical analysis to show the quantitative variability. The replicates were grouped into technical replicates (TRs) and biological replicates (BRs) (Fig 1) to perform statistical tests. For graphical representation and analysis, Microsoft excel and R-studio with R-version 3.4.3 were used. Venn diagrams and heatmaps for qualitative data were plotted with the LFQ values (S1–S3 Tables) comparing all 9 Rs. The heatmaps for quantitative data were plotted with the APEX values to compare TRs and BRs. To generate data for heatmaps a reference list was made by pooling the protein abundance values from all the 9 Rs in decreasing order of abundance. On the other hand, to filter the topmost abundant proteins, an arbitrary cut-off of 2.0E6 was considered for all the 3 cell lines. The APEX abundance values of the 9 Rs per cell line were averaged, and abundance of 2.0E6 and above was considered the most abundant. The arbitrary cut-off was set based on user-defined (1.0E8 in our data) normalization factor C, which is an estimate of the total protein abundance in 1 sample.

The variance of TRs and BRs was calculated in terms of relative standard deviation (RSD) [22, 40]. RSD was calculated using the following equation:

$$RSD=\frac{SD}{\bar{x}}\times 100$$

Where SD is the standard deviation of a dataset, and $\bar{x}$ is the mean value of a dataset. SD was calculated using the following equation:

$$SD=\sqrt{\frac{\sum_{i=0}^{n}{\left(x_{i}-\bar{x}\right)}^{2}}{n-1}}$$

Where x_i_ is the observed value of 1 sample item, $\bar{x}$ is the mean value of the observations, and n is the total number of observations.

The RSD of LFQ values in TRs was calculated as the percentage of the SD of the number of proteins identified in R1, R2, R3 / R4, R5, R6 / R7, R8, R9 divided by the average number of proteins identified in R1, R2, R3 / R4, R5, R6 / R7, R8, R9 and used as a numeric representation of the technical variance. RSD of LFQ values in TRs was calculated as follows:

$$RSD_{LFQ\_TRs}=\frac{\sqrt{\frac{\sum_{i=0}^{n}{\left(\left(TR_{i}-\bar{TRs}\right)\right)}^{2}}{n-1}}}{\bar{TRs}}\times 100$$

Where TR_i_ is the observed value of the sum of total proteins identified in each TR (i.e, ∑TR_1_, ∑TR_2_, and ∑TR_3_), $\bar{TRs}$ is the mean value of the proteins identified in 3 TRs, and n is the total number of observations.

Whereas the RSD in BRs was calculated as the percentage of the SD of number of proteins identified in R1, R4, R7 / R2, R5, R8 / R3, R6, R9 divided by the average number of proteins identified in R1, R4, R7 / R2, R5, R8 / R3, R6, R9 and used as a numeric representation of the biological variance. RSD of LFQ values in BRs was calculated as follows:

$$RSD_{LFQ_{-}BRs}=\frac{\sqrt{\frac{\sum_{i=0}^{n}{\left(\left(BR_{i}-\bar{BRs}\right)\right)}^{2}}{n-1}}}{\bar{BRs}}\times 100$$

Where BR_i_ is the observed value of the sum of total proteins identified in each BR (i.e, ∑BR_1_, ∑BR_2_, and ∑BR_3_), $\bar{BRs}$ is the mean value of the proteins identified in 3 BRs, and n is the total number of observations.

The RSD of APEX values in TRs was calculated as the percentage of the SD of the protein abundance in R1, R2, R3 / R4, R5, R6 / R7, R8, R9 divided by the average of protein abundance in R1, R2, R3 / R4, R5, R6 / R7, R8, R9 and used as a numeric representation of the technical variance. RSD of APEX values in TRs was calculated as follows:

$$RSD_{APEX\_TRs}=\frac{\sqrt{\frac{{\left((\sum TR_{1}-\bar{TRs}\right)}^{2}+{\left(\sum TR_{2}-\bar{TRs}\right)}^{2}+{\left(\sum TR_{3}-\bar{TRs}\right)}^{2}}{n-1}}}{\bar{TRs}}\times 100$$

Where ∑TR_1_ is the observed value of the sum of the protein abundances of ∑R_1_, ∑R_2_, and ∑R_3_, ∑TR_2_ is the observed value of the sum of the protein abundances of ∑R_4_, ∑R_5_, and ∑R_6_, ∑TR_3_ is the observed value of the sum of the protein abundances of ∑R_7_, ∑R_8_, and ∑R_9_, $\bar{TRs}$ is the mean value of the proteins identified in 3 TRs, and n is the total number of observations.

Whereas the RSD in BRs was calculated as the percentage of the SD of the protein abundance in R1, R4, R7 / R2, R5, R8 / R3, R6, R9 divided by the average of protein abundance in R1, R4, R7 / R2, R5, R8 / R3, R6, R9 and used as a numeric representation of the biological variance. RSD of APEX values in BRs was calculated as follows:

$$RSD_{APEX\_BRs}=\frac{\sqrt{\frac{{\left((\sum BR_{1}-\bar{BRs}\right)}^{2}+{\left(\sum BR_{2}-\bar{BRs}\right)}^{2}+{\left(\sum BR_{3}-\bar{BRs}\right)}^{2}}{n-1}}}{\bar{BRs}}\times 100$$

Where ∑BR_1_ is the observed value of the sum of the protein abundances of ∑R_1_, ∑R_4_, and ∑R_7_, ∑BR_2_ is the observed value of the sum of the protein abundances of ∑R_2_, ∑R_5_, and ∑R_8_, ∑BR_3_ is the observed value of the sum of the protein abundances of ∑R_3_, ∑R_6_, and ∑R_9_, $\bar{BRs}$ is the mean value of the proteins identified in 3 BRs, and n is the total number of observations.

One-way repeated measures analysis of variance (ANOVA) was conducted to check for statistically significant differences in the means of proteins levels of TRs, BRs, and the 9 Rs of all the cell lines. A *p*-value of less than 0.016 was held as the threshold for identifying significant changes among TRs and BRs by applying the standard Bonferroni [41, 42] correction (α/3 = 0.05/3) considering 3 groups. A *p*-value of less than 0.0055 was held as the threshold for identifying significant changes between 9 Rs by applying the standard Bonferroni correction (α/9 = 0.05/9) considering a total of 9 groups.

Power analysis was performed on the total proteins identified in the 9 Rs per cell line using the power ANOVA test in the R-statistical package. This was performed at a significance level of 0.016, and a power of 0.8 to identify how many Rs will be needed to confidently identify all possible EE-EV proteins in the 3 cell lines. Further, the power analysis was applied to determine how many more folds of proteins would be obtained relative to the effect size when using 9 Rs.

## Results

### Replicates generation

Triplicate EE-EV samples from lung adenocarcinoma cell lines H1993, A549 and H1975 generated at different passages, here referred as A, B and C, were used to generate technical replicates (TRs) and biological replicates (BRs) as shown in Fig 1. Altogether, 27 replicate samples (Rs), representing 9 Rs per cell line, were analyzed for this study.

### Identification of exosomal markers

CD9 and CD81 were identified by LC/MS/MS in EE-EV Rs from all 3 cell lines, while TSG101 was detected in Rs from cell line H1993. The presence of these and other exosomal markers such as CD63, Flotillin 1, and Calnexin was confirmed by western blot analysis (S1 Fig).

### Particle size distribution of EE-EVs

The minimum and maximum diameters of vesicles isolated from H1993 were 82 nm and 656 nm, respectively, with a mean diameter of 157 + 73.3 nm and a d90 value of 249 nm (90% of the vesicles showed a diameter below 249 nm) (S2 Fig). The minimum and maximum diameters of vesicles isolated from A549 were 65 nm and 564 nm respectively, with a mean diameter of 145 + 82.3 nm, and a d90 value of 249 nm (90% of the vesicles showed a diameter below 249 nm) (S2 Fig). The minimum and maximum diameters of vesicles isolated from H1975 were 63 nm and 560 nm, respectively, with a mean diameter of 146 + 76.8 nm, and a d90 value of 231 nm (90% of the vesicles showed a diameter below 231 nm) (S2 Fig). These diameters are consistent with that of exosomes with a small microvesicle contamination, as known to happen in exosome isolations [6, 43].

### Qualitative variability analysis

**Fig 2:**
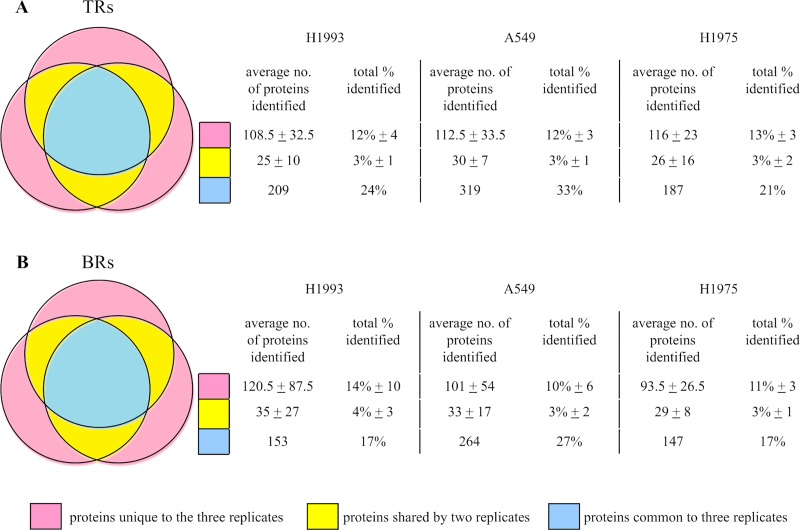
Venn diagrams and tables showing the average number of proteins (in total and in percentage of the total) identified across three TRs and three BRs out of the total proteins detected in the three cell lines H1993 (886), A549 (976) and H1975 (879), respectively for (A) TRs (B) BRs. The proteins unique to the three replicates is shown in pink, the proteins shared by 2 replicates is shown in yellow, and the proteins common to three replicates is shown in blue.

The EE-EV proteins identified in triplicates of TRs and BRs from each of the 3 cell lines (S3 and S4 Figs) were subjected to qualitative variability analysis (Fig 2) The average qualitative variability from each of the 3 TRs and BRs, the average number of proteins unique to each R, shared by 2 Rs, and common to 3 Rs was calculated. Venn diagrams and tables were used to show the average number of proteins (in total and in percentage of the total) identified across 3 TRs and 3 BRs out of the total proteins detected in the 3 cell lines H1993 (886), A549 (976) and H1975 (879), respectively for TRs (Fig 2A) and for BRs (Fig 2B). These results indicated an average 6% higher qualitative variability in BRs than in TRs for the 3 cell lines studied.

**Fig 3:**
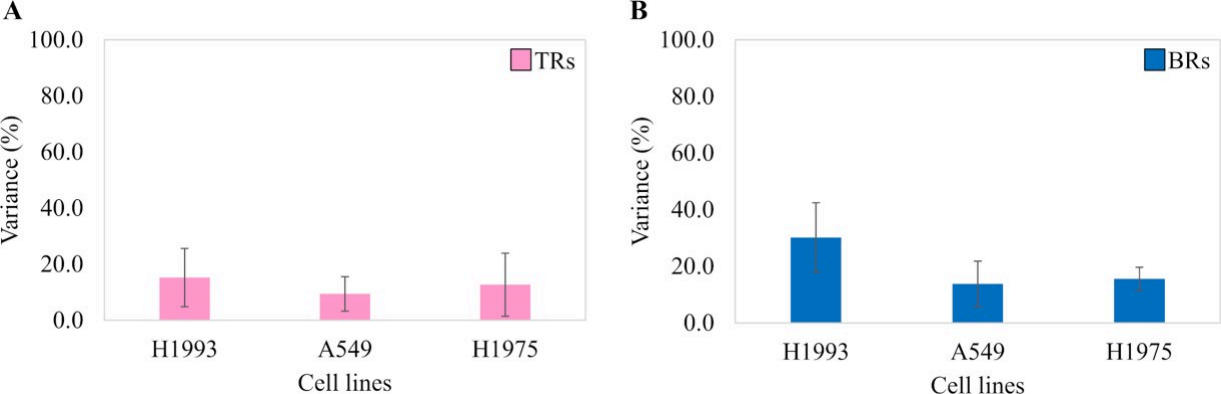
Variance analysis showing qualitative variability in TRs and BRs of three H1993, A549, and H1975 cell line exosomes.

Variance analysis showed qualitative variability in 3 cell line EE-EVs TRs and BRs (Fig 3A and 3B, respectively). The RSD values in BRs of H1993 (30.2%), A549 (13.8%) and H1975 (15.6%) EE-EVs can be observed to be higher than in their respective TRs (H1993–15.2%, A549–9.4% and H1975–12.7%). Therefore, RSD analysis of the qualitative data also indicated that compared to TRs (Fig 3A), BRs (Fig 3B) showed a higher variance for all the 3 cell lines studied (7%).

**Fig 4:**
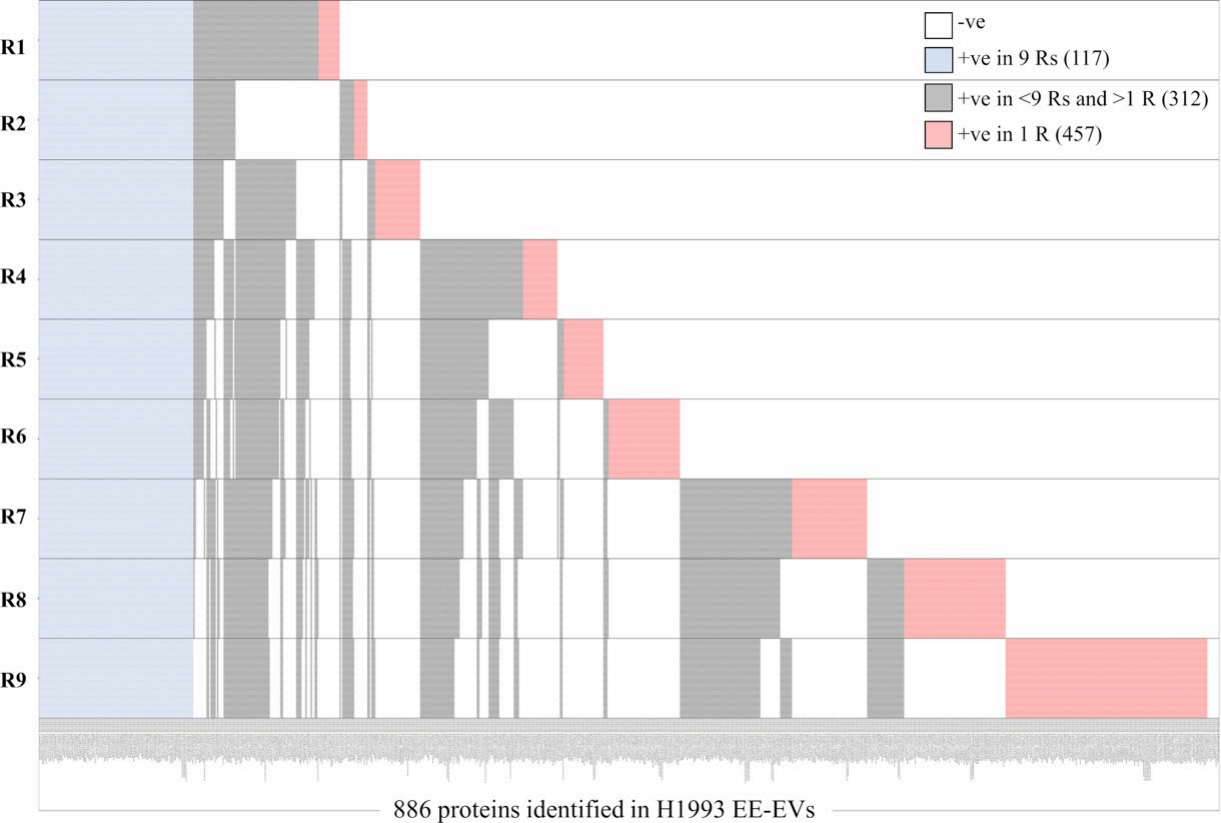
Heatmap showing number of samples in which each of the total 886 proteins was identified in H1993 exosomes. Proteins common to all nine replicates are shown in blue (117). Proteins present in >1 replicate and <9 replicates are shown in grey (312) and proteins present only in 1 replicate are shown in red (457).

**Fig 5:**
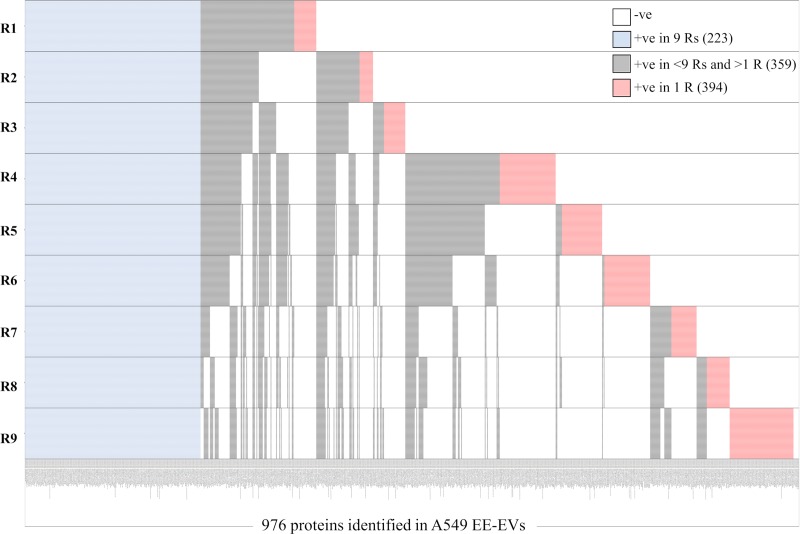
Heatmap showing number of samples in which each of the total 976 proteins was identified in A549 exosomes. **Proteins common to all nine replicates are shown in blue (223).** Proteins present in >1 replicate and <9 replicates are shown in grey (359) and proteins present only in 1 replicate are shown in red (394).

**Fig 6:**
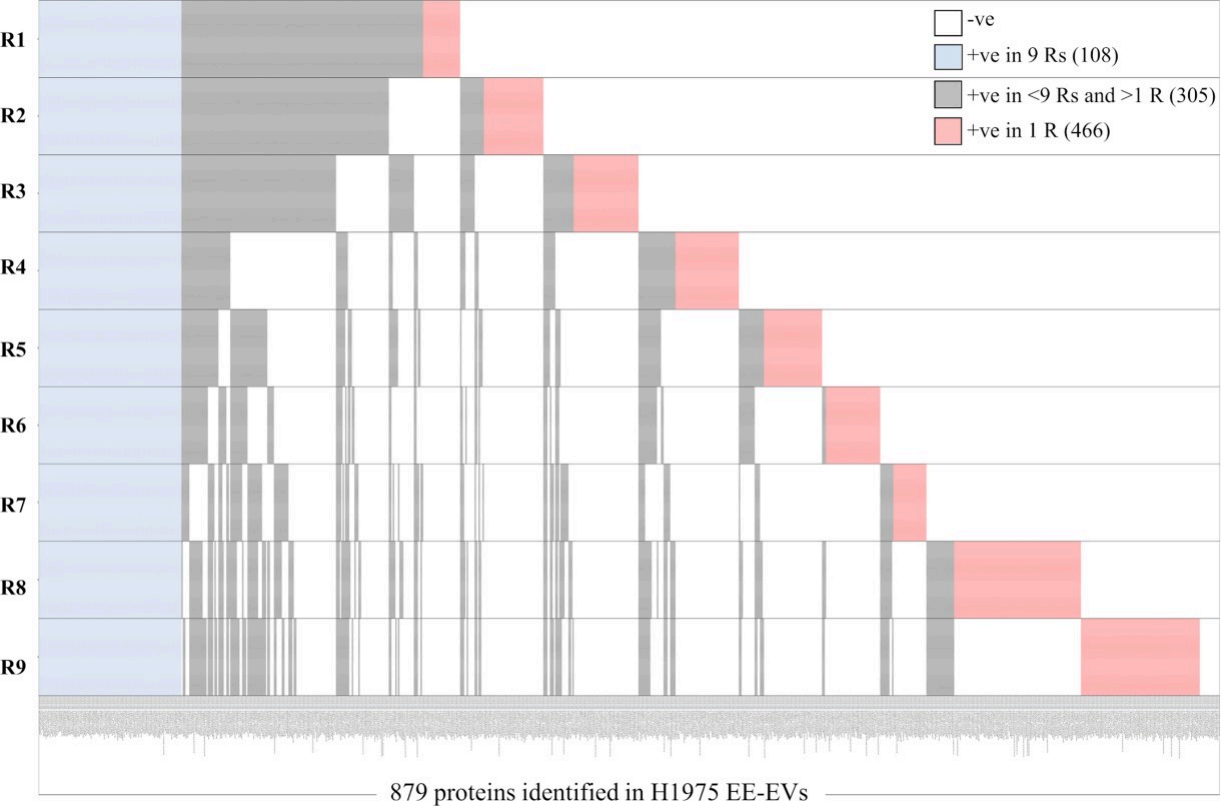
Heatmap showing number of samples in which each of the total 879 proteins was identified in H1975 exosomes. Proteins common to all nine replicates are shown in blue (108). Proteins present in >1 replicate and <9 replicates are shown in grey (305) and proteins present only in 1 replicate are shown in red (466).

In addition, qualitative heatmaps of all the proteins identified in the 9 Rs (+ve in 9 Rs) obtained from each cell line indicated that a high number of proteins identified in a single R (+ve in 1 R) were absent (-ve) in the other 2, whereas the number of proteins in common among the 9 Rs was relatively small (S1–S3 Tables and Figs 4–6). Heatmap for a total of 886 proteins identified in 9 Rs of H1993 EE-EVs (Fig 4) showed 117 proteins common to all 9 Rs (+ve in 9 Rs), 312 proteins present in >1 R and <9 Rs (+ve in <9 Rs and >1 R) and 457 proteins present only in 1 R (+ve in 1 R). The number of proteins identified only in 1 R (457) was higher than those identified in all 9 Rs (117) together. Heatmap for a total of 976 proteins identified in 9 Rs of A549 EE-EVs (Fig 5) showed 223 proteins common to all 9 Rs (+ve in 9 Rs), 359 proteins present in >1 R and <9 Rs (+ve in <9 Rs and >1 R) and 394 proteins present only in 1 R (+ve in 1 R). Therefore, also in this second cell line the number of proteins identified only in 1 R (394) was higher than those identified in all 9 Rs (223) together. Heatmap for a total of 879 proteins identified in 9 Rs of H1975 EE-EVs (Fig 6) showed 108 proteins common to all 9 Rs (+ve in 9 Rs), 305 proteins present in >1 R and <9 Rs (+ve in <9 Rs and >1 R) and 466 proteins present only in 1 R (+ve in 1 R). Hence, in the third cell line, the number of proteins identified only in 1 R (466) was higher than those identified in all 9 Rs (108) together. Therefore, all the 9 Rs, obtained per cell line contributed to the completeness of the EE-EV profile in each of the 3 cell lines studied.

### Quantitative variability analysis

**Fig 7:**
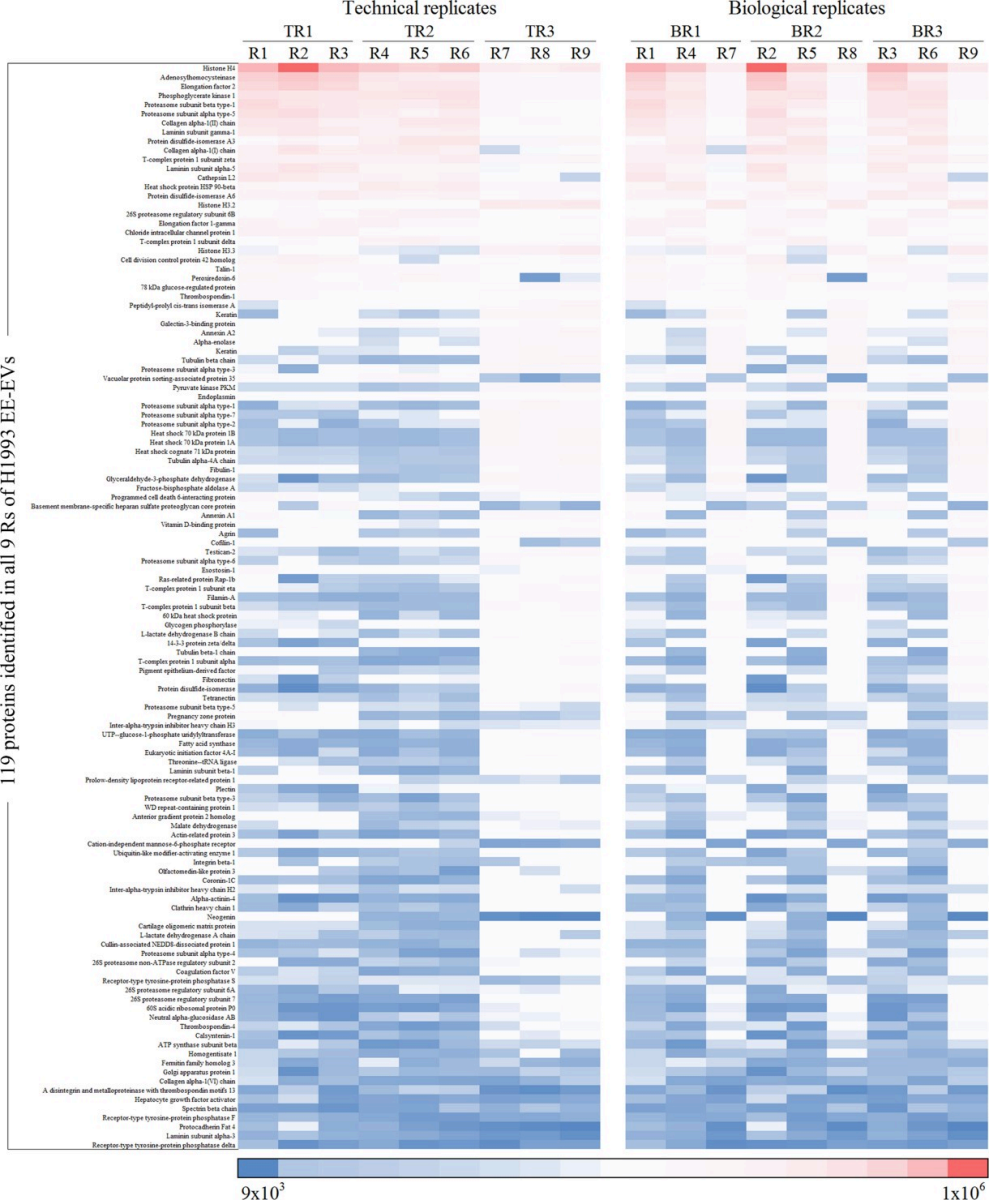
Heatmap representing exosomal proteins identified in H1993 in order of decreasing levels in TRs (R1-R2-R3, R4-R5-R6, R7-R8-R9) and in BRs (R1-R4-R7, R2-R5-R6, R3-R6-R9). Boxes highlighted in dark red represent the most abundant, while boxes in dark blue represent the least abundant proteins.

**Fig 8:**
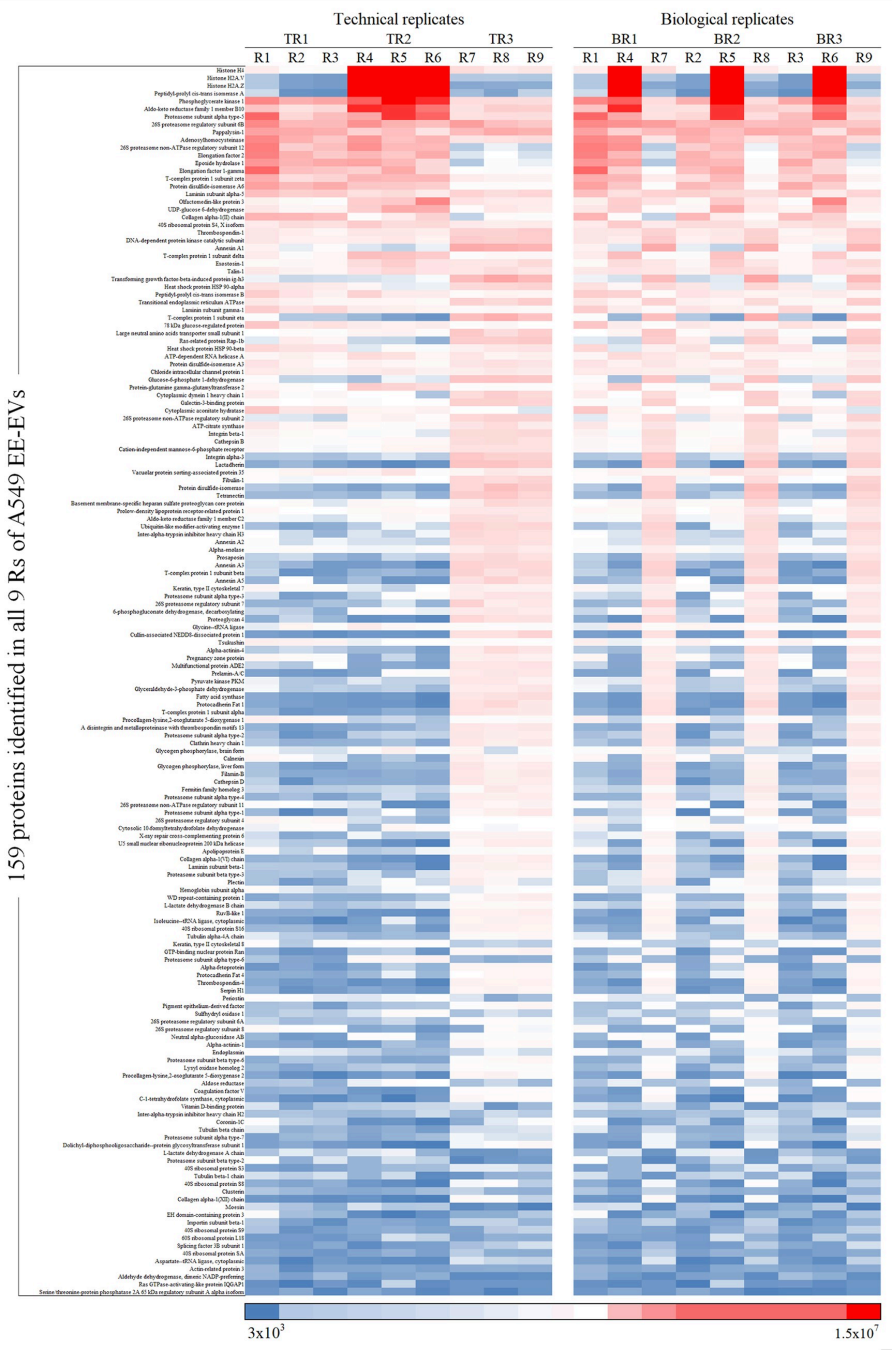
Heatmap representing exosomal proteins identified in A549 in order of decreasing levels in TRs (R1-R2-R3, R4-R5-R6, R7-R8-R9) and in BRs (R1-R4-R7, R2-R5-R6, R3-R6-R9). Boxes highlighted in dark red represent the most abundant, while boxes in dark blue represent the least abundant proteins.

**Fig 9:**
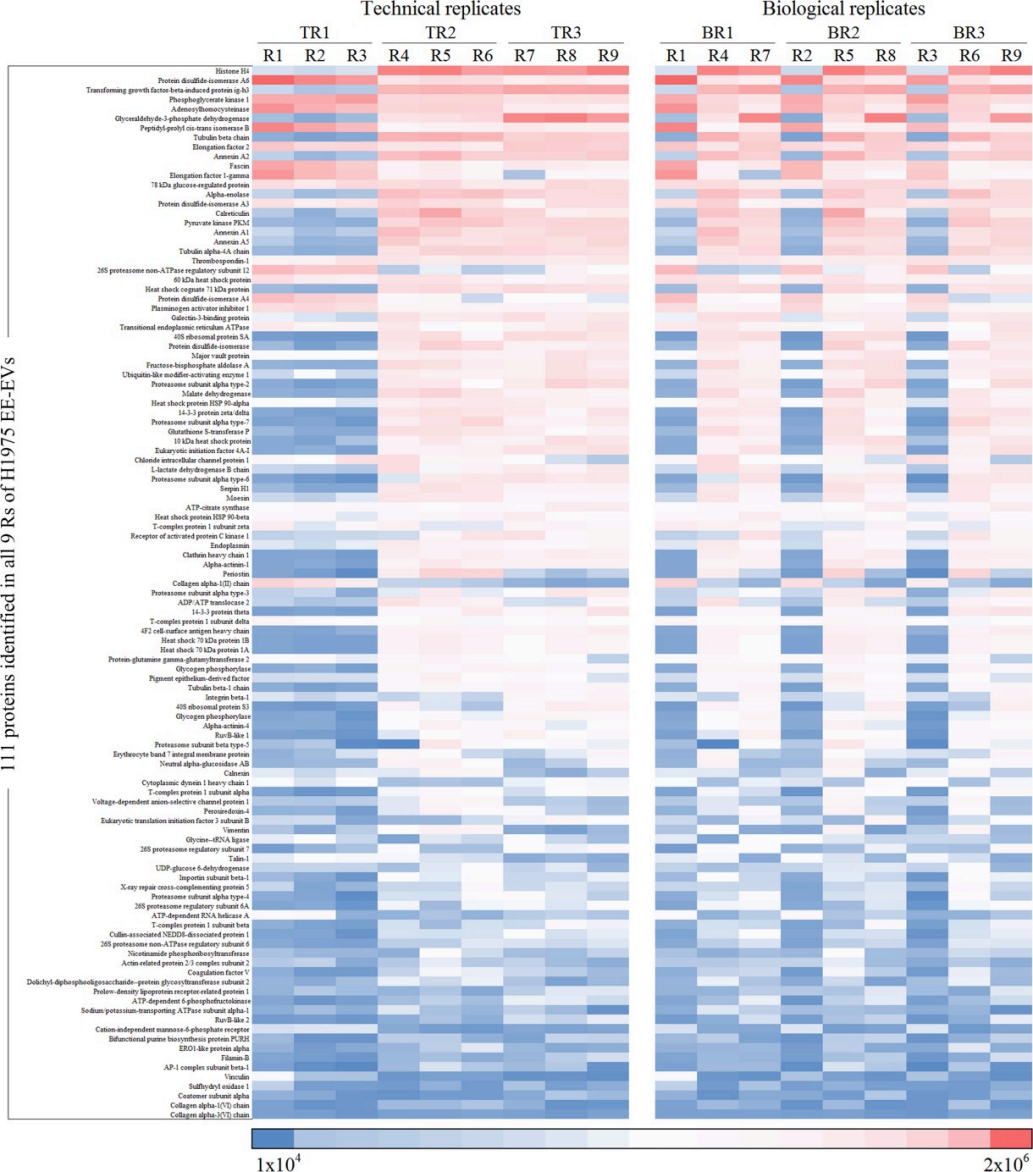
Heatmap representing exosomal proteins identified in H1975 in order of decreasing levels in TRs (R1-R2-R3, R4-R5-R6, R7-R8-R9) and in BRs (R1-R4-R7, R2-R5-R6, R3-R6-R9). Boxes highlighted in dark red represent the most abundant, while boxes in dark blue represent the least abundant proteins.

Heatmaps were used to show the quantitative variability among exosomal proteins identified in triplicates of TRs and BRs from the 3 cell lines studied (Figs 7–9). EE-EVs proteins identified in H1993 (Fig 7), A549 (Fig 8) and H1975 (Fig 9) are shown in the order of decreasing levels in TRs (R1-R2-R3, R4-R5-R6, R7-R8-R9) as well as in BRs (R1-R4-R7, R2-R5-R6, R3-R6-R9). From these heatmaps, it can be concluded that the abundance of each protein was similar across all the Rs. However, the BRs showed more quantitative variability compared to TRs. The APEX values for each of the Rs are presented in S4 Table.

**Fig 10:**
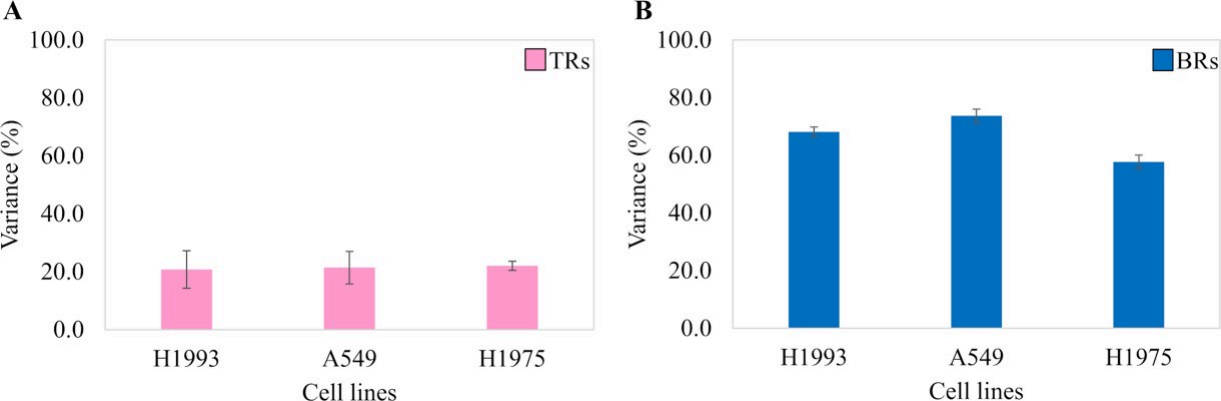
Variance analysis showing quantitative variability in TRs and BRs of three H1993, A549, and H1975 cell line exosomes.

To evaluate the variance between Rs, we assessed the quantitative variability by RSD analysis of the quantitative data from all the 3 cell line EE-EVs TRs and BRs (Fig 10A and 10B, respectively). This figure (Fig 10) shows that the RSD values in BRs of H1993 (68.2%), A549 (75.8%) and H1975 (59.8%) EE-EVs were higher than in their respective TRs (H1993–20.8%, A549–21.4% and H1975–22.0%). In conclusion, RSD analysis indicated that compared to TRs (Fig 10A), BRs (Fig 10B) showed 47% higher variance in BRs than in TRs for all the 3 cell lines studied.

However, there was no statistically significant quantitative variability observed among the triplicates of TRs and BRs, except for the H1975 cell line (Table 1), which showed statistically significant quantitative variability in TRs, BRs as well as all the 9 Rs. All these data show a cumulatively higher degree of variability in Rs of H1975 cell line EE-EVs when compared to the H1993 and A549 cell line EE-EVs.

**Table 1:** Table shows one-way repeated measures ANOVA F-values and corresponding *p*-values for the levels of proteins identified in EE-EVs in 3 TRs and 3 BRs for H1993, A549 and H1975 cell lines.

**A.**		
Sample	One-way repeated ANOVA	
	F—value	*p*—value
H1993 TRs	2.2	0.113
A549 TRs	2.35	0.097
H1975 TRs	11.38	1.98E-005**
**B.**		
Sample	One-way repeated ANOVA	
	F—value	*p*—value
H1993 BRs	1.891	0.153
A549 BRs	2.923	0.0552
H1975 BRs	6.413	0.00916*
**C.**		
Sample	One-way repeated ANOVA	
	F—value	*p*—value
H1993 9 Rs	2.373	0.0156
A549 9 Rs	2.385	0.015
H1975 9 Rs	10.99	7.37E-15***

* represents *p* < 0.016, which is considered statistically significant. Table 1A represents the analysis of TRs. Table 1B represents the analysis of BRs, and Table 1C represents the analysis of 9 Rs.

### Power analysis

Finally, we assessed how much the number of EE-EVs proteins would increase if we analyzed all the Rs indicated by the power analysis, which was 18, 27 and 252 Rs for cell lines H1993, A549 and H1975, respectively. We found that the analysis of all these Rs would only result in 0.2 fold increase in the number of proteins identified in EE-EVs from cell line H1993, 0.3 fold increase in the number of proteins from cell line A549 and 0.4 fold increase in the number of proteins from the cell line H1975. Therefore, increasing the number of Rs would not increase significantly the number of total proteins identified in their EE-EVs.

## Discussion

The study of exosomal cargo often requires high-throughput analysis of replicate samples (Rs). As the number and abundance of identified biomolecules varies between Rs, establishing the replicate variability predicted for the event under study is essential in determining the number of Rs required for reaching accurate conclusions. Since, to the best of our knowledge, the variability between Rs of any of the various types of exosomal cargo has not been previously reported; in this study, we used LC/MS/MS analysis of exosome enriched EVs (EE-EVs) technical replicates (TRs) and biological replicates (BRs) from 3 different lung adenocarcinoma cell lines to determine the qualitative and quantitative variability in the detected proteins. To maximize protein identification, we analyzed 100 μg of total exosomal protein per replicate [44].

Our workflow started by establishing the qualitative variability among Rs. Venn diagrams and RSD analysis showed considerable variability in the proteins identified in TRs; an unexpected finding considering that each set of TRs originates from a single EE-EV sample. Therefore, technical factors have an impact on protein identification by LC/MS/MS analysis, which cannot be overlooked. Among the technical sources of TRs variability, including extraction, digestion, instrumental variance and instrumental stability, a study conducted by Piehowski et al concluded that the main source of variability is instrumental variance, and mainly involves ion-suppression and chromatographic disturbances [45].

The qualitative variability among BRs was, in average, 6% higher than that of TRs for the 3 cell lines studied, pointing to the existence of a small set of passage-dependent proteins. In vivo, changes in exosomal protein cargo occur due to a variety of causes, including viral infections [46], internal diseases [47–49], radiation [50], and ageing [51]. Interestingly, when we generated a heatmap of the proteins present in each of the 9 Rs obtained per cell line, we found that the number of proteins unique to each replicate was higher than the number of proteins common to all 9. Therefore, each of the Rs, whether TRs or BRs, contributed to generate a more complete EE-EVs protein profile. This finding had an impact on the power analysis, which is discussed in a later paragraph.

Next, we focused on the quantitative analysis of the data. Heatmaps showed that the abundance of each protein was similar across all the Rs. Although, the BRs showed more variability than the TRs, a finding previously reported for quantitative LC/MS/MS [52]. Our results, however, stood in contrast with a cell metabolome study in which the variability in BRs was found to be lower than that of the TRs [22]. Such a discrepancy between studies suggests that variability among BRs may hinge on the biological system under study, with some more stable than others. With regard to the additional biostatistical analyses performed here, the RSD analysis supported what we observed in the quantitative heatmaps, although it showed a 47% higher variance in BRs than in TRs for all the 3 cell lines studied, which is also likely to be a passage-dependent effect. The ANOVA analysis, however, showed no statistically significant differences in protein abundance among Rs, except for the H1975 cell line.

Importantly, for all 3 cell lines studied, the abundance of the 90% top proteins was similar in BRs and TRs, an observation consistent with the general concept in mass spectrometry studies that the top 75% most abundant proteins in Rs from a complex sample are very reproducibly detected, but the bottom 25% are quite variable [53].

We finally determined the power analysis for each of the 3 cell lines studied based on the 9 Rs collected from each one. Such analysis indicated that 23 Rs were required to identify the maximum number of EE-EV proteins in H1993 and A549 cell lines, and approximately a 10-fold higher number of Rs was required for the H1975 cell line, which showed the highest qualitative and quantitative variability between Rs. These numbers were expected based on the qualitative heatmaps previously discussed, and for the first 2 cell lines it was slightly below of n = thirty, which statisticians consider appropriate to get a feeling for the mean and its distribution [21]. Nevertheless, the generation of as many Rs as indicated by our power analysis, is unrealistic, both for practical and financial reasons. Therefore, it is important to stress that performing all these Rs will only produce a 0.3-folds increase in EE-EV protein detection for all the 3 cell lines studied.

In conclusion, we found that the variability among TRs as well as BRs was largely qualitative and higher among BRs. By contrast, the quantitative variability was low, except for a single cell line where the quantitative variability was significant. Importantly, our replicate strategy of analyzing 3 BRs, each divided into 3 TRs, identified 90% of the most abundant proteins, thereby establishing the utility of our approach.

## Supporting information

S1 Fig Western blot showing the presence of exosomal markers in EE-EVs lysate (EL) and total cell lysate (TCL) obtained from H1993, A549 and H1975 cell lines. (TIF)

S2 Fig TRPS analysis of EE-EVs from H1993, A549 and H1975 cell lines. (TIF)

S3 Fig Venn diagrams showing the number of proteins identified in 3 EE-EV TRs from the total proteins detected in the 3 cell lines H1993 (886), A549 (976) and H1975 (879), respectively. (TIF)

S4 Fig Venn diagrams showing the number of proteins identified in 3 EE-EV BRs from the total proteins detected in the 3 cell lines H1993 (886), A549 (976) and H1975 (879), respectively. (TIF)

S1 Table LFQ intensity values for all the 9 Rs in H1993 EE-EVs for checking presence/ absence of proteins. (XLS)

S2 Table LFQ intensity values for all the 9 Rs in A549 EE-EVs for checking presence/ absence of proteins. (XLS)

S3 Table LFQ intensity values for all the 9 Rs in H1975 EE-EVs for checking presence/ absence of proteins. (XLS)

S4 Table APEX values of the list of proteins identified in common across nine replicates in H1993, A549 and H1975 exosomes. Protein names represented in Figs 7–9 are given here.
(XLS)
