# Supplementary figures and images for: Variability in protein cargo detection in technical and biological replicates of exosome-enriched extracellular vesicles
Source: PLoS One. 2020 Mar 2;15(3):e0228871. doi: 10.1371/journal.pone.0228871 (PMC7051218; doi:10.1371/journal.pone.0228871)

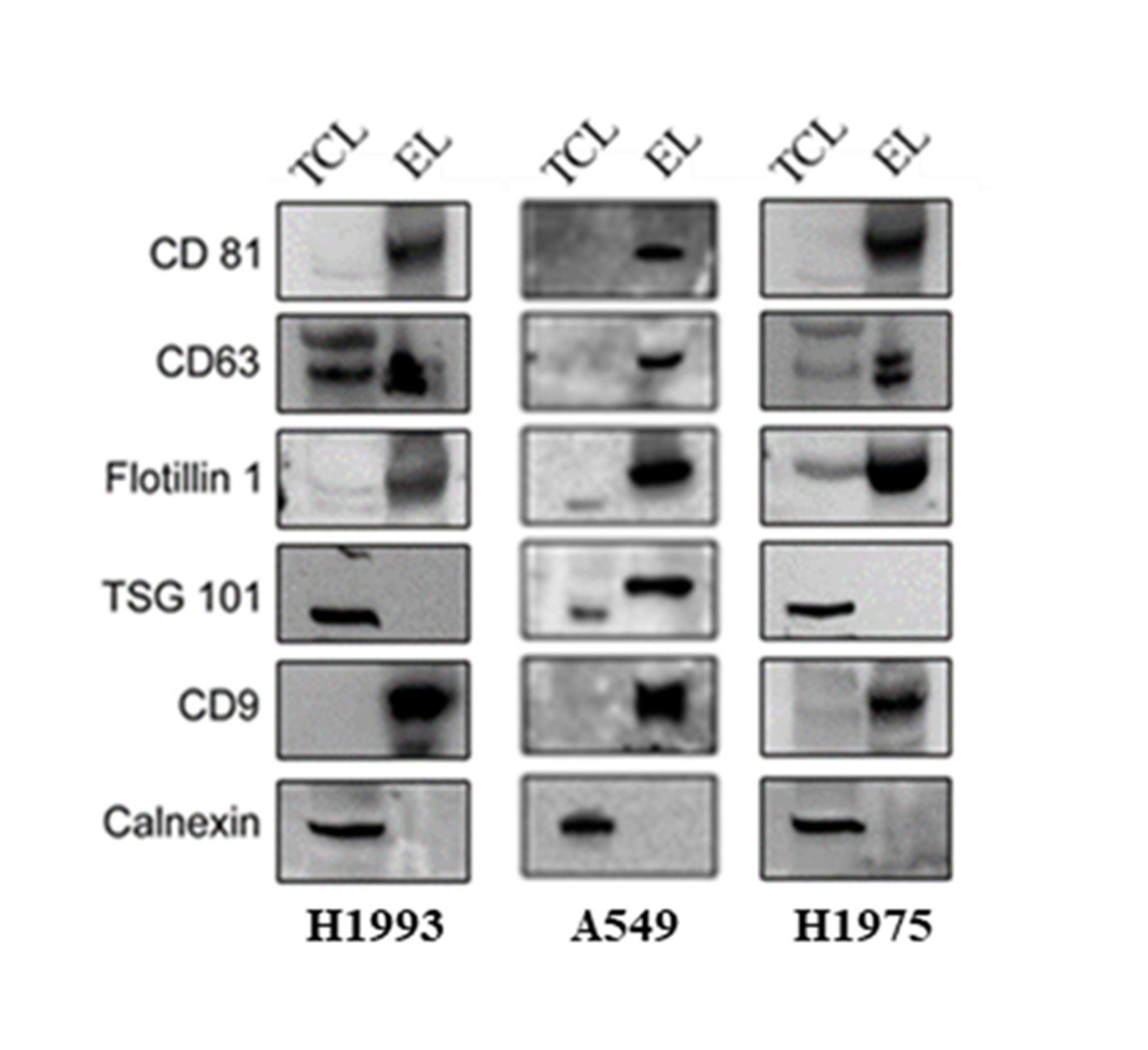

Supplement: S1 Fig — (TIF) [file pone.0228871.s001.tif]

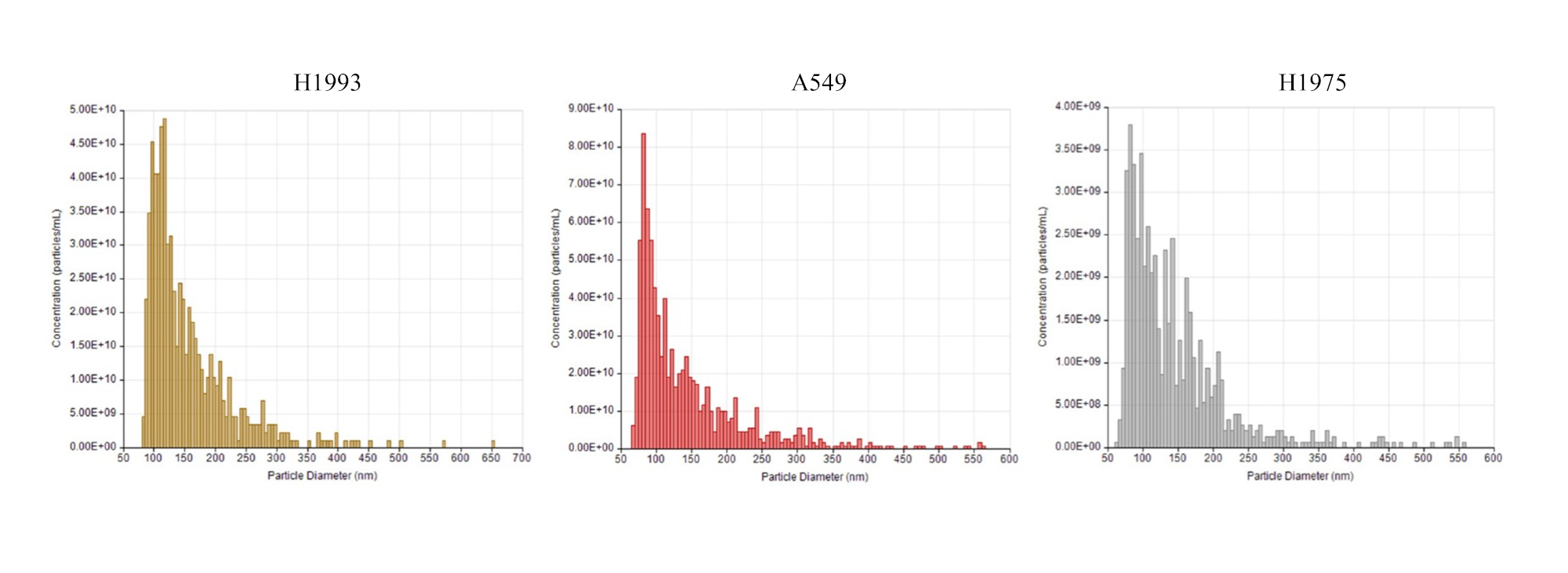

Supplement: S2 Fig — (TIF) [file pone.0228871.s002.tif]

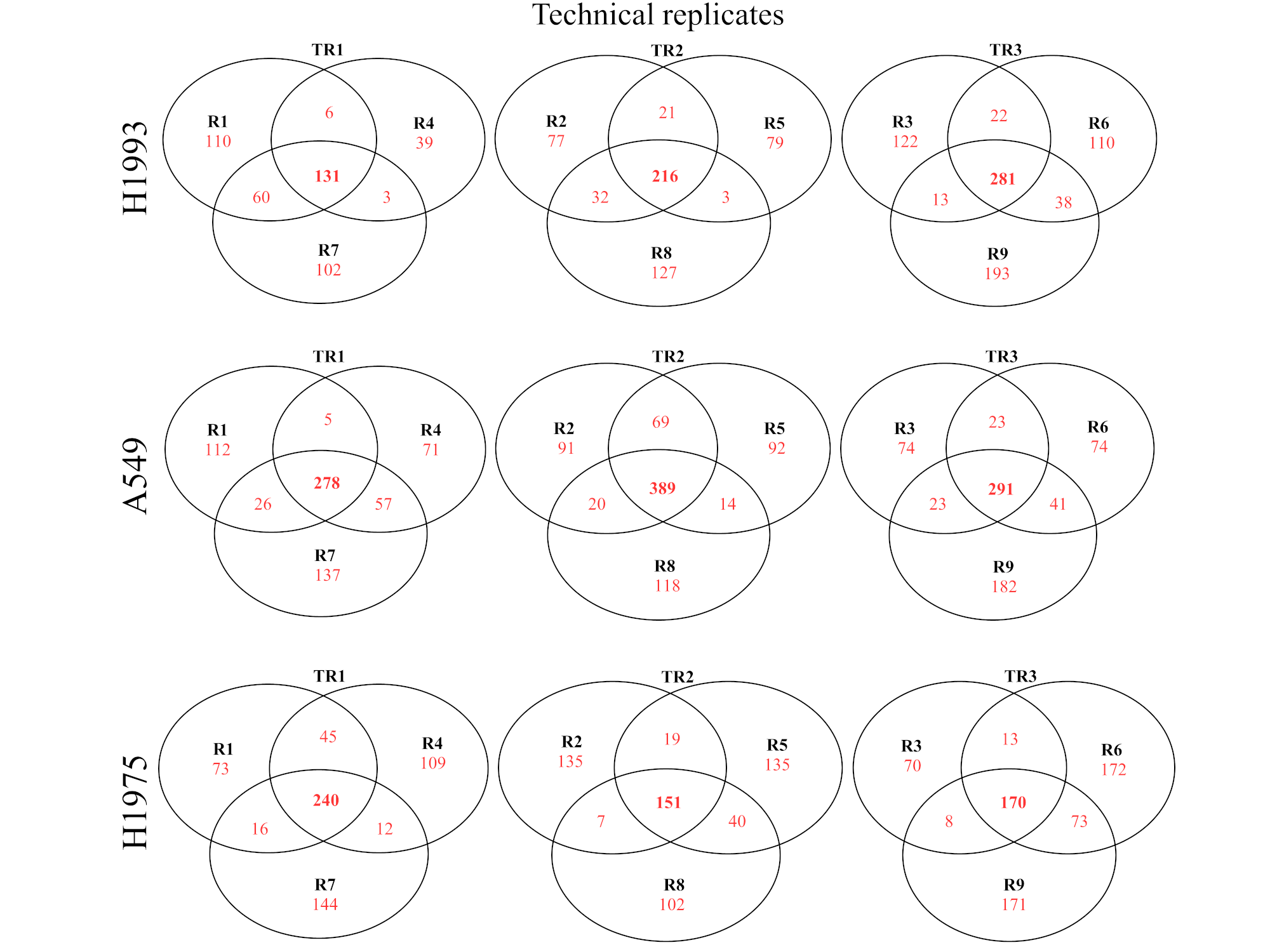

Supplement: S3 Fig — (TIF) [file pone.0228871.s003.tif]

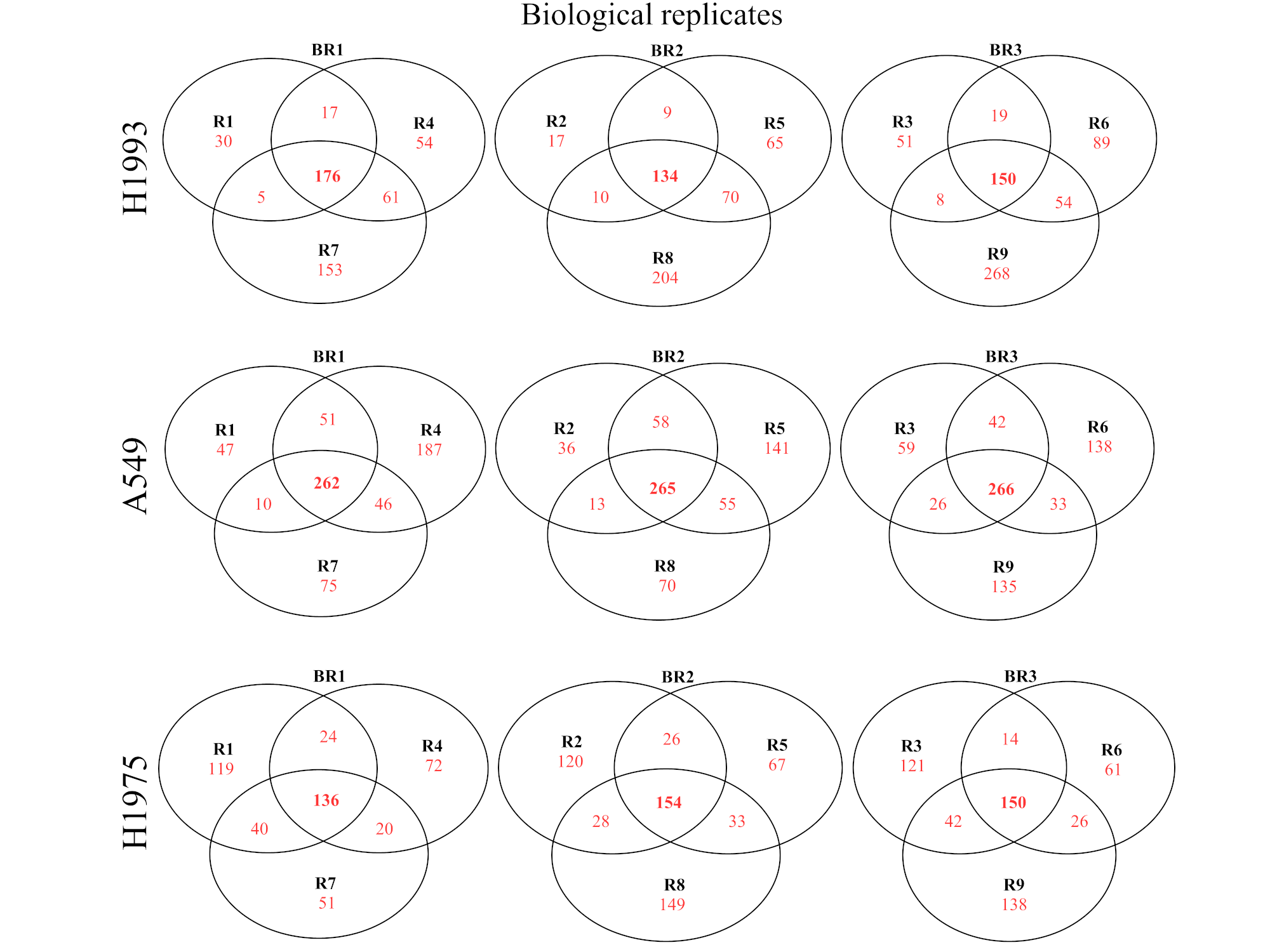

Supplement: S4 Fig — (TIF) [file pone.0228871.s004.tif]
